# Interruptions in nutritional therapy in children with chronic diseases during the acute phase of critical illness and their effect on the administered volume

**DOI:** 10.3389/fnut.2025.1548574

**Published:** 2025-04-30

**Authors:** Patrícia Zamberlan, Petrovane Morais de Torres, Juliana Caires De Oliveira Achili Ferreira, Werther Brunow Carvalho, Artur Delgado

**Affiliations:** Division of Nutrition, Instituto da Criança e do Adolescente/Hospital das Clínicas, Faculty of Medicine, University of São Paulo, São Paulo, Brazil

**Keywords:** nutrition therapy, enteral nutrition, parenteral nutrition, child hospitalized, intensive care units, pediatric, nutrients

## Abstract

**Introduction:**

Undernutrition is a common concern among critically ill children and adolescents in the hospital setting. Inadequate delivery of nutrition therapy (NT) can significantly contribute to the prevalence of undernutrition in pediatric intensive care units (PICUs).

**Objective:**

To assess the impact of interruptions on the volume of NT delivered to children with basal chronic conditions, as well as the calories and proteins provided, during the acute phase in the PICU.

**Methods:**

A cohort of critically ill pediatric patients with basal chronic conditions was analyzed to evaluate the administration of enteral or parenteral nutrition. The focus was on the volume of NT not effectively delivered and the underlying causes of incomplete NT.

**Results:**

A total of 120 children were included, the majority of whom were undernourished (47.5%) by body mass index-for-age (BMI/A). A significant proportion of the prescribed enteral (EN) or parenteral nutrition (PN) was not administered during the first and third days of evaluation. The most prevalent reasons for interruptions were procedures delays and complications arising during treatment in the PICU.

**Conclusion:**

Interruptions in NT delivery are frequent among critically ill children; the volume of NT not delivered was greater on the third day for both EN and PN after initiation; procedures delays and mild gastrointestinal complications were the main causes of incomplete NT administration.

## Introduction

Undernutrition is defined as a nutrient intake imbalance resulting in cumulative energy, protein or micronutrient deficit and consequent negative impact on the growth/development and the immune system with higher risk of infections. Sarcopenia and several kinds of infections can occur, including pneumonia associated with mechanical ventilation (MV) (1–4). In recent years, several scientific studies have described the repercussions of insufficient nutrition therapy (NT) delivery as associated with worse morbidity/mortality – deterioration of nutritional status, poor wound healing, reduced free days of MV, longer stay in the hospital, lower bed turnover, and higher costs of inpatient treatment and for the health system as a whole (5–7).

Hospital undernutrition is common in developing and low-income countries. Approximately 20 to 50% of adult inpatients exhibit undernutrition (8–10) and in low-income countries according to some reports, undernutrition affects 51% of the hospitalized pediatric population (11). Critically ill patients are highly predisposed to develop undernutrition, which might be present in 20 to 30% of children admitted to pediatric intensive care units (PICUs) (12, 13), with a mortality rate varying from 9 to 38% (3). Therefore, it can be assumed that many children admitted to the PICU, especially those with chronic diseases, will develop some additional degree of undernutrition (14). Nutrients administration to children under MV, for example, is frequently inadequate: some reports showed that only 38 and 43% of the prescribed goal of proteins and calories were administered, respectively. Deficits in NT volume impact the total nutritional intake, which, in turn, may indirectly affect the recovery and progression of critically ill children (11, 15, 16).

Evidence regarding the impact of nutritional support during critical illness on short- and long-term outcomes is mixed and inconclusive. Mehta et al. (17) found that a higher percentage of prescribed dietary energy via the enteral route is associated with improved 60-day survival. However, the TARGET trial found no significant difference in mortality or quality of life at 6 months between critically ill patients receiving 100% versus 70% of their enteral calorie requirements, suggesting that delivering full caloric intake does not necessarily improve long-term outcomes, and recent findings suggest that early high-dose nutrition may be harmful in critically ill patients, potentially due to the suppression of cellular repair pathways and anabolic resistance (18). This underscores the complexity of nutritional support in critical care and the need for individualized approaches (19).

Despite the association between undernutrition during and after a PICU stay and poorer outcomes, it is important to highlight that there is no evidence that nutritional support during critical illness improves short- or long-term outcomes (20, 21). Traditionally, nutritional interventions are initiated during the acute phase of critical illness, which is then followed by stable and recovery phases. These three phases are marked by evolving neuroendocrine, immunologic, and metabolic responses over time (22) and these phase-specific changes necessitate different macronutrient intakes.

The acute phase of critical illness in children is characterized by the requirement of (escalating) vital organ support and nutritional influences the acute stress response. However, contrary to earlier beliefs, increased nutrient provision during this phase seems not to reverse hypercatabolism or the resulting muscle atrophy (23) and cumulative evidence from large randomized controlled trials (RCTs) revealing harm by providing full nutrition in the acute phase (24).

However, there is still debate regarding the impact of nutrition adequacy of critical illness phases, especially in the acute phase, it is important to monitor interruptions and their impact on the supply to ensure adequate nutritional provision during the stay in the PICU, thus preventing the worsening of nutritional status and its consequences. Studying interruptions in NT during the acute phase in the PICU is essential because this period is critical for patient stabilization and can directly impact recovery. Although there are studies on NT in critically ill children, there is little specific evidence on how interruptions affect the final volume administered, particularly during this early phase. Our study aimed to fill this gap by quantifying volume related these interruptions and hypothesizing that they may compromise planned nutritional intake, which in turn could indirectly influence clinical outcomes (25). We believe that a better understanding of this phenomenon can contribute to strategies that minimize nutritional loss and optimize NT in critically ill pediatric patients.

Therefore, the main objective of this study was to evaluate the impact of interruptions on the volume of NT offered to children, as well as on the calories and proteins provided, during the early acute phase (particularly during the first 5 days of admission) in the PICU, when patients are generally on mechanical ventilation and use vasoactive drugs.

## Methods

This prospective study was conducted at the clinical-surgical PICU of a university hospital in the city of São Paulo, Brazil, in a tertiary care facility, during a 2-years period. This 15-bed, tertiary PICU primarily attends to patients from 2 months–18 years (until the month before the 19th birthday) with chronic diseases. On average, 90% of the children admitted to this unit have chronic diseases, 70% require mechanical ventilation, and 65% need one or more vasoactive drugs.

### Study population

This prospective study included children, between the ages of 6 months and 5 years (to achieve a greater homogeneity within the study population), who were admitted to the PICU, in general with a chronic disease (at least 3 months) (26), for any type of treatment, who remained in the PICU for more than 48 h and who received NT [enteral nutrition therapy (EN) and/or parenteral nutrition therapy (PN)] and who had nutritional NT initiated within the first 24 to 72 h of admission. Children with congenital or acquired malformations of the gastrointestinal system, history of diarrhea and/or vomiting in the past 30 days, cases of readmission to the PICU and patients transferred from other units to PICU (to minimize the bias of them receiving or having already received NT before admission to PICU) during the study period were excluded.

Sample size calculations were conducted with a 5% margin of error and a 95% confidence interval in mind. We estimated an admission rate of 40 patients per month, with data collection planned to span 24 months. Additionally, we considered that approximately 35 to 40% of the children admitted to our PICU are older than 5 years. We also accounted for a potential loss of up to 10% in enteral nutrition volume and 5% in parenteral nutrition volume.

To characterize the sample, the following data were collected: gender, age, mortality, discharge, mortality risk score, nutrition status, PICU length of stay (LOS) and time to onset of NT.

### Anthropometric assessment

Anthropometric nutrition assessment was performed within 24 h of admission to characterize the study population and it included weight, height or length, and body mass index (BMI).

Anthropometric nutritional evaluation was carried out within 24 h of admission always by the same professional. The measurement of the weight was performed with a scale calibrated for accuracy before each measurement. For children < 2 years the weight was obtained on a baby scale. For children > 2 years who had clinical condition the weight was measured standing on a digital platform scale. Those children who could not be weighed standing (intense sedation, compensated shock, mechanical ventilation, etc.) were held by an adult. The weight of the child was obtained by subtracting the weight of the adult from the total weight (adult plus child) as described. When a child presented with severe edema, we relied on the habitual weight provided by the child’s caregiver prior to admission to the PICU. This choice was made to prevent any interference with the interpretation of anthropometric measurements and the calculation of nutritional requirements. By doing so, we aimed to minimize potential distortions in our nutritional assessments.

In children aged < 2 years the length was measured using a pediatric anthropometer with an accuracy of 0.1 cm. In children > 2 years was utilized a wooden stadimeter for height with an accuracy of 0.1 cm. In children with difficulties to perform conventional measuring techniques (hemodynamic instability, under sedation, mechanical ventilation, etc.), height was predicted from measurements of the distance between the knee and ankle using the technique proposed by Chumlea et al. (27) or by tibia length as proposed by Stevenson et al (28). Using the data for weight and height or length, we obtained BMI with the following equation: weight (kg)/height (2) (m). Anthropometric classification on admission was defined by the BMI-for-age (BMI/A) z-score using the reference values from the World Health Organization (WHO) (29): undernutrition (BMI z-score <−2), eutrophic (BMI z-score ≥ − 2 and ≤ + 1), overweight (BMI z-score > + 1 and ≤ + 2), or obese (BMI z-score> + 2).

### Nutrition therapy

**Figure 1:**
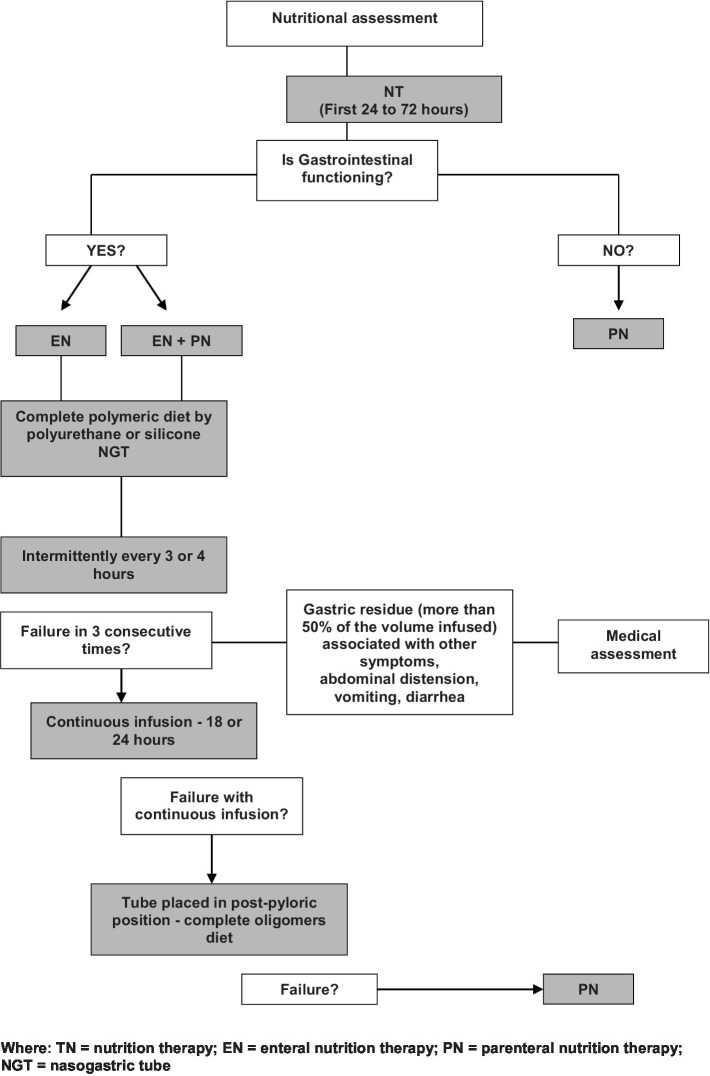
NT flow chart (30).

We initiated NT after hemodynamic stabilization (we aimed to initiate NT within the first 24–72 h of admission) and EN was preferably indicated and prioritized, while PN was used only when the digestive tract could not be utilized. When transitioning from PN to EN, we adhered to the protocol established by the hospital’s NT team (Figure 1) (30). In our unit, we have an established approach for feeding while on vasopressors and we follow the flowchart provided by recommendations for clinicians caring for children (including infants, school-aged children, and adolescents) with septic shock and other sepsis-associated organ dysfunction (31).

The caloric requirements of the patients were estimated using the Schofield equation (weight and height) (32). We started with 30% of the energy requirements and progressively increasing daily according to tolerance, aiming for a minimum of 60% of the required energy by the third day. Following to the recent American Society Parenteral and Enteral Nutrition (ASPEN) guidelines (33), we started with the target of 1.5 g/kg/day and we plan to make adjustments according to the patient’s needs during hospitalization, based on the clinical progression.

In general, we used complete polymeric diets adequate to age by gastric route (hydrolyzed diet as needed) and personalized prescription of PN for children and adolescents (30). We considered the polymeric diet as a type of EN that contains macronutrients in their intact form, such as whole proteins, carbohydrates, and fats and they are often used in clinical settings for patients who require nutritional support but have a functioning gastrointestinal tract capable of digesting and absorbing complex nutrients. In contrast, an oligomeric diet, often referred to as an elemental or semi-elemental diet, consists of nutrients in their simplest form, such as oligopeptides or amino acids, simple sugars, and medium-chain triglycerides. These diets are designed to be easily absorbed in the gastrointestinal tract with minimal digestive effort, making them suitable for patients with compromised digestive function.

The prescribed and administered volume, calories and proteins (enteral and/or parenteral) were recorded on the first and third day after the introduction of NT.

EN and PN was always administered through infusion pumping by the nursing staff charged with evaluating the prescribed and effectively administered volume. The daily effectively infused volume was compared to the prescribed volume, and the not administered volume was calculated. Causes for possible NT interruption in the period were analyzed.

Data collection on NT assessment were based on information from printed/electronic medical records, medical prescriptions, care plans, nursing notes and nutrition assessments.

### Assessment of severity

The patients had the severity of their clinical conditions evaluated by the Pediatric Index of Mortality (PIM) score (34), which is based on 8 clinical variables collected at the time of admission and is useful to evaluate patient severity.

### Statistical analysis

The results were tabulated using Excel (Microsoft®). Statistical analysis was performed using JAMOVI software, version 2.3 (2022). Comparisons of the variables—prescribed volume on the first and third days versus non-administered volume on the first and third days—were conducted using the Wilcoxon W test after verifying the normality of the data distribution (Shapiro–Wilk test).

Nominal variables were described as frequencies and continuous variables were described as the median and Interquartile range (IQR) when non- normally distributed data.

## Results

**Figure 2:**
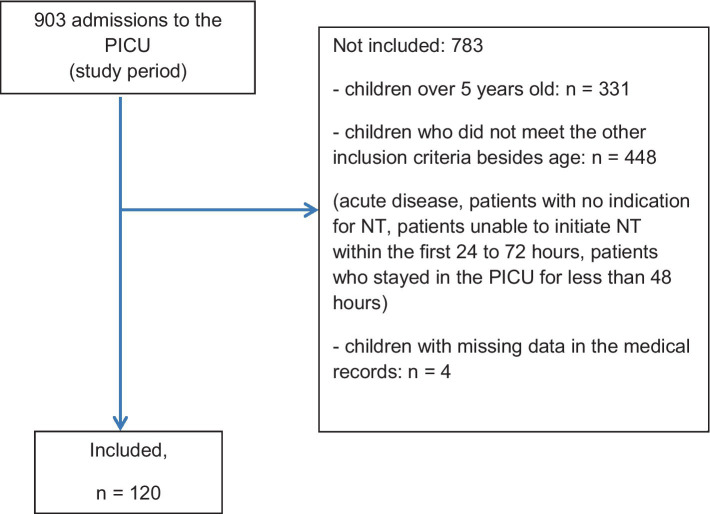
Flowchart—main characteristics of the study population.

Among the 903 patients admitted to the PICU during the study period, 779 patients were excluded as they did not meet the inclusion criteria. Following secondary screenings, 4 patients were excluded from the 124 (Figure 2). The study population comprised 120 patients.

The median age of studied population was 9 months, 90% of them had chronic diseases with acute complication and 17 (14.1%) deaths occurred during hospitalization in the PICU. The main diagnosis on admission was respiratory diseases and 86.7% of these patients required MV. The demographic characteristics of the studied population are described in Table 1.

**Table 1:** Demographic characteristics of the studied population.

Variable	Median (IQR) or %
Age (months)	9.0 (4–17)
Gender	
Male	54.2
Female	45.8
Nutritional status	
Well nourished	20.8
Undernourished = 47.5%;	47.5
Overweight = 7.5%;	7.5
Obesity = 5.9%	5.9
Diagnosis category	
Respiratory diseases	58.3
Liver diseases	16.6
Other diseases (genetic syndromes, neurological diseases, nephropathies, oncological diseases, post-operative conditions)	25.1
Time onset of NT	
≤ 48 h	81.7
> 48 h until 72 h	18.3
PIM	2.1 (2–92)
Mortality	14.1
PICU length of stay (days)	11.0 (3–140)

NT, nutritional therapy; IQR, Interquartile range.

### Nutrition therapy

Of the 120 patients included in the study, 108 received EN and 12 received PN, corresponding to 90 and 10%, respectively.

Table 2 presents the main data related to NT.

**Table 2:** Data related to NT.

Variable	Day 1 (%)	Day 3 (%)
Type of diet		
Polymeric	95.4	96.3
Oligomeric	4.6	3.7
Route of administration		
Gastric	97.3	97.3
Nasogastric tube	100	100
Gastrostomy	0	0
Jejunal	2.7	2.7
Mode of EN administration		
Bolus (infusion pump)	97.3	97.3
Continuous	2.7	2.7
EN		
Patients who had volume interruptions	28.7 (93.5% = MV, 87.1% = sedation, 22.6% = one or more vasoactive drugs)*	30.5 (78% = MV, 60% = sedation, 12.1% = one or more vasoactive drugs)*
PN		
Patients who had volume interruptions	91.6	100
Reasons for non-administration of EN		
Extubation	29.0	30.3
Nausea/vomiting	12.9	15.2
Abdominal distension	12.9	18.2
Gastric residue**	6.5	3.0
Other (radiographic exams and other medical procedures, patient transport, changes in mechanical ventilation parameters, transient hemodynamic instability, and diarrhea)	38.7	33.3
Reasons for non-administration of PN		
Interruption for medication administration	79.2	79.2
Break for therapeutic procedures	12.5	12.5
Other (delay in PN infusion, delay in PN bag replacement, catheter-related issues (e.g., obstruction, need for replacement), transient clinical instability)	8.3	8.3

*Clinical features about the patients. **Gastric residue (GR) was considered the presence of 50% or more of the volume prescribed and infused during the past 4 h. For intermittent of gastric NT, GR was considered the presence of 50% or more of the prescribed volume that was assessed immediately before the onset of the following feeding. EN, enteral nutrition; PN, parenteral nutrition; MV, mechanical ventilation.

The comparative analysis between the prescribed and not administered volume on the first and third day for both EN and PN. There was a significant difference, with higher non-administered volumes on the third day for both EN and PN as described in Table 3.

**Table 3:** Comparative analysis between the prescribed and not administered volume, calorie and protein (day 1 and 3) for EN and PN.

	Day 1 Median (%) (IQR)	Day 3 Median (%) (IQR)	*p*
EN			
*Volume (ml)*			
Prescribed	400 (100) (260–640)	560 (100) (400–840)	< 0.001
Not administered	70 (17.5) (39–150)	110 (19.6) (60–195)	< 0.001
*Calorias (Kcal/dia)*			
Prescribed	300 (100) (203–532)	400 (100) (280–710)	< 0.001
Not administered	56 (18.6) (27.5–115)	88 (22.0) (46–159)	< 0.001
*Protein (g/dia)*			
Prescribed	5.5 (100) (3–10)	7.0 (100) (4–14)	< 0.001
Not administered	1.0 (18.2) (0.1–2)	1.0 (14.3) (0.1–3)	< 0.001
PN			
*Volume (ml)*			
Prescribed	510 (100) (475–903)	591 (100) (472–826)	< 0.001
Not administered	34 (6.7) (22.5–59.5)	87.5 (14.8) (44.8–200)	< 0.001

EN, enteral nutrition; PN, parenteral nutrition; IQR, Interquartile range.

## Discussion

Children with chronic critical illness represent growing populations with high healthcare use and dependence on specialized care, both in the hospital and community setting (35). Nutrition assessment and delivery NT in the PICU during the acute phase of critical clinical condition (especially in first and third day of introduction) is crucial for improving clinical outcomes (in short and long-term) in children with chronic diseases related to malnutrition, which is highly prevalent in this population (17, 36, 37). Interruptions in nutritional therapy during this phase of illness, particularly in critically ill patients, can have significant and lasting effects on recovery, long-term nutritional status, and overall health outcomes beyond the hospital stay (impact on quality of life and post-intensive care syndrome, recovery and functional outcomes, long-term nutritional disorders) (38–41).

Despite the ongoing debate regarding the impact of NT adequacy during this phase of critical illness in PICU, the primary goals include: (A) provision of adequate energy and protein in order to ensure that critically ill children receive sufficient energy and protein for supporting their metabolic needs and promoting recovery. The literature emphasizes the importance of achieving at least 60% of the prescribed energy and protein targets within the first week of PICU admission, as this is associated with lower mortality rates (33); to preferably use the enteral route (EN) for nutrient delivery in patients with a functioning gastrointestinal tract. It is associated with improved survival, reduced infection rates, and better maintenance of gut integrity compared to PN; to initiate EN early which helps to mitigate the negative energy balance that often occurs during the acute phase of critical illness. This early intervention supports organ function restoration and reduces the risk of complications associated with delayed nutrition; avoidance of early PN that has been associated with increased infection rates and longer PICU stays (41). Therefore, delaying PN during the first week of critical illness, especially in patients who can tolerate some level of EN, is recommended to avoid these adverse outcomes; tailoring NT to the individual needs of each patient is essential. This includes accurate assessment of energy requirements and careful monitoring of daily energy and protein intake to adjust the nutrition plan as needed; and minimizing interruptions in EN that can lead to inadequate nutrient delivery (17, 33, 36, 37, 42–45).

In the context of minimizing interruptions to ensure the delivery of nutrients, the difference between the volume prescribed and the volume infused is a significant issue. The medical literature highlights several factors contributing to this discrepancy. Critically ill children often receive less than the prescribed amount of EN due to various interruptions and clinical challenges. A study by Iglesias et al. found that more than 50% of enteral nutrition days involved a delivered-to-required energy ratio of less than 90% (35). The primary reasons for this underdelivery included clinical instability, airway management, radiologic and surgical procedures, and accidental feeding tube removal. Additionally, the administration of vasoactive drugs was independently associated with a lower energy supply (46). Mehta et al. also reported that EN was interrupted on average for at least 2 days in 71% of patients, leading to a mean daily nutritional intake of only 38% of the prescribed energy goals (17).This underdelivery of nutrition was associated with higher mortality rates and a higher prevalence of acquired infections, emphasizing the clinical significance of the gap between prescribed and delivered nutrition.

In our study 90% of the patients used the enteral route for nutrition. Several interruptions were observed due to food intolerance (nausea, vomiting, abdominal distension) and procedures such as extubation. This resulted in a failure to infuse the planned volume by more than 20% on the first day after the introduction of EN and more than 30% by the third day, corroborating the cited studies. In the study by Mehta et al., the interruptions were also often due to clinical procedures, perceived feeding intolerance, and other factors that could potentially be mitigated. The study highlighted that achieving a higher percentage of the prescribed energy intake via the enteral route was significantly associated with lower 60-day mortality, emphasizing the importance of minimizing interruptions to optimize clinical outcomes (17). Besides, interruptions can lead to significant deficits in nutritional intake, which is crucial for maintaining adequate nutritional status and subsequent recovery and reducing the risk of complications.

Most of our patients initiated EN within 48 h of admission, but interruptions affected the total enteral volume delivered in the first days. Nevertheless, these patients likely received approximately 60% of their prescribed energy and protein requirements during the first week in the PICU. By the third day, nearly 70% of the targeted volume had been administered, aligning with literature recommendations to minimize cumulative energy deficits, which may be linked to adverse clinical and nutritional outcomes (33).

The current consensus, as reflected in the medical literature, suggests that early PN should generally be avoided in the first week of critical illness in children, unless there are specific contraindications to EN or significant malnutrition that cannot be addressed otherwise (33, 44, 47). The PEPaNIC trial demonstrated that withholding PN during the first week in critically ill children led to a reduction in new infections and accelerated recovery compared to early PN administration. This trial also found that early withholding of PN did not negatively impact long-term outcomes such as mortality, growth, or neurocognitive development, and in fact, improved neurocognitive outcomes (41).

In our study, 10% of the patients used PN. The parenteral infused volume was even lower than the enteral volume, probably due to the clinical instability of these patients, as the main cause for the interruption was the administration of medications - absence of an exclusive route for the administration of PN. The administration of PN alongside drugs in the same catheter is a complex issue that requires careful consideration of compatibility and safety. The simultaneous administration of medications with PN admixtures can lead to pharmacological incompatibility, which may affect the stability of the PN emulsion. This can result in complications such as precipitation in the infusion line or catheter occlusion, potentially leading to serious adverse events like embolization or inflammatory reactions (17, 33). Therefore, the European Society for Clinical Nutrition and Metabolism (ESPEN) and the American Society for Parenteral and Enteral Nutrition (ASPEN) generally advise against including non-nutrient drugs in PN admixtures due to these safety concerns (33). The recommendation is to use multi-lumen catheters, reserving one lumen exclusively for PN. However, there are circumstances where co-administration via a Y-site may be considered, provided that compatibility has been thoroughly evaluated.

The strengths of this study include its prospective design and the involvement of a sample of patients with chronic diseases admitted to a tertiary-level PICU at a national reference center, where EN/PN losses were evaluated by a specialized nutrition support team. However, the study’s limitations include the heterogeneity of the patient sample, its single-center design, and the absence of sequential assessments of the patients’ nutritional status. Moreover, we did not distinguish the adequacy of energy and protein intake based on different nutritional status categories (undernourished, eutrophic, overweight) or severity that would provide insights into whether undernourished children received appropriate compensatory intake or if nutrient provision was uniform across all categories.

Despite the limitations of our study, it demonstrates that numerous interruptions occur in the provision of NT during the acute phase of critical illness, leading to a reduction in the volume of NT delivered. This is an important aspect because, although the acute phase focuses on the adequate provision of proteins and careful administration of calories, monitoring the non-infused volume is necessary to prevent long-term nutritional deficits, especially in children with chronic diseases.

Our results allow us to conclude that interruptions in the administration of NT (whether enteral or parenteral) had an impact on the final infused volume. Interruptions in NT are common in the PICU and can significantly impact the volume of nutrition actually delivered compared to what is prescribed. These interruptions may be due to various clinical procedures, feeding intolerance, or other clinical decisions (17, 33). The use of vasoactive drugs, for example, is one of the critical factors. While there is a concern that vasoactive medications might affect gastrointestinal perfusion and thus the safety of EN, studies have indicated that enteral feeding can be safely administered in critically ill children receiving these medications without adverse effects (33). However, the presence of these drugs may still influence clinical decisions regarding the initiation and continuation of EN, potentially leading to reduced volumes being infused compared to what is prescribed.

Volume-based feeding (VBF) strategies have been proposed to address the issue of underfeeding by allowing adjustments in the infusion rate to compensate for interruptions, thereby improving the delivery of the prescribed nutritional volume (48–50). These strategies have been shown to increase the percentage of goal calories delivered without increasing adverse outcomes, suggesting that they can be an effective approach to bridging the gap between prescribed and infused volumes (48–50).

Overall, the discrepancy between prescribed and infused volumes in critically ill children is multifactorial, involving clinical practices, patient-specific factors, and the implementation and continuous review of feeding protocols. Addressing these factors through strategies like VBF and minimizing avoidable interruptions can help optimize nutrition delivery in this vulnerable population.

## Data Availability

The original contributions presented in the study are included in the article/supplementary material, further inquiries can be directed to the corresponding author.
